# Prediction of Fetal Death in Preterm Preeclampsia Using Fetal Sex, Placental Growth Factor and Gestational Age

**DOI:** 10.3390/jpm14101059

**Published:** 2024-10-13

**Authors:** Blanca Novillo-Del Álamo, Alicia Martínez-Varea, Carmen Sánchez-Arco, Elisa Simarro-Suárez, Iker González-Blanco, Mar Nieto-Tous, José Morales-Roselló

**Affiliations:** 1 Department of Obstetrics and Gynaecology, La Fe University and Polytechnic Hospital, Avenida Fernando Abril Martorell 106, 46026 Valencia, Spain; martinez_alivar@gva.es (A.M.-V.); sanchez_cararc@gva.es (C.S.-A.); simarro_eli@gva.es (E.S.-S.); gonzalez_ike@gva.es (I.G.-B.); nieto_martou@gva.es (M.N.-T.); jose.morales@uv.es (J.M.-R.); 2 Department of Medicine, CEU Cardenal Herrera University, 12006 Castellón de la Plana, Spain; 3 Department of Pediatrics, Obstetrics and Gynecology, Faculty of Medicine, University of Valencia, 46010 Valencia, Spain

**Keywords:** preeclampsia, fetal sex, placental growth factor, perinatal death, gestational age

## Abstract

Background/Objectives: Preeclampsia (PE) is a systemic disease that affects 4.6% of pregnancies. Despite the existence of a first-trimester screening for the prediction of preterm PE, no consensus exists regarding neither the right moment to end the pregnancy nor the appropriate variables to estimate the prognosis. The objective of this study was to obtain a prediction model for perinatal death in patients with preterm PE, useful for clinical practice. Methods: Singleton pregnant women with PE and preterm delivery were included in an observational retrospective study. Multiple maternal and fetal variables were collected, and several multivariable logistic regression analyses were applied to construct models to predict perinatal death, selecting the most accurate and reproducible according to the highest area under the curve (AUC) and the lowest Akaike Information Criteria (AIC). Results: A group of 148 pregnant women were included, and 18 perinatal deaths were registered. Univariable logistic regression selected as statistically significant variables the following: gestational age (GA) at admission, fetal sex, poor response to antihypertensive drugs, PlGF, umbilical artery (UA) pulsatility index (PI), cerebroplacental ratio (CPR), and absent/reversed ductus venosus (DV). The multivariable model, including all these parameters, presented an AUC of 0.95 and an AIC of 76.5. However, a model including only GA and fetal sex presented a similar accuracy with the highest simplicity (AUC 0.93, AIC 67.6). Finally, in fetuses with a similar GA, fetal death became dependent on PlGF and fetal sex, underlying the role of fetal sex in all circumstances. Conclusions: Female fetal sex and low PlGF are notorious predictors of perinatal death in preterm PE, only surpassed by early GA at birth.

## 1. Introduction

Preeclampsia (PE) is a multisystem disease characterized by the appearance of hypertension after the 20th week of pregnancy, associated with a widespread organic dysfunction [1,2,3,4,5] that produces significant maternal–fetal comorbidities and has long-term implications on women’s lives. PE is a cardiovascular risk enhancer [6], affecting 4.6% of pregnancies, although this incidence has recently increased [1,2].

Screening for preterm PE is performed in the first trimester of pregnancy [7]. Women with a high risk of PE must initiate acetylsalicylic acid 150 mg daily until week 36 to reduce the possibility of adverse perinatal and maternal outcomes [8,9,10]. During their pregnancy, exhaustive surveillance (analytical, clinical, and ultrasonographical) is carried out to detect the onset of the disease early. However, not infrequently, PE is diagnosed when organ failures have started and consequences in the fetus have already occurred [4,11].

The pathophysiology of PE lies in placental vascular dysfunction [11]. Based on this fact, multiple studies have analyzed the usefulness of the PE ratio sFlt-1/PlGF [12,13,14], agreeing on the high sensitivity and negative predictive value for the development of PE in the subsequent weeks [12,13,14,15]. However, no consensus has been reached, neither about including the ratio as a diagnostic criterion for PE [3,15,16,17,18,19,20] nor about including the ratio to pinpoint the right moment to end the pregnancy in cases of severe PE.

The objective of our study was to evaluate which factors influence perinatal death, creating a prediction model for perinatal death in patients with preterm PE.

## 2. Materials and Methods

This was an observational retrospective study that included preterm singleton pregnancies diagnosed with PE who attended the Obstetric Department of the tertiary maternity center Hospital Universitario y Politécnico La Fe between 2013 and 2023.

The inclusion criteria were singleton pregnancies diagnosed with PE with a complete follow-up and a preterm delivery. The exclusion criteria were twins, term pregnancies, and pregnancies with loss of follow-up. The Hospital Research Ethics Committee approved the study on the 29 March 2023 (register number: 2023-217-1).

The variables were collected via reviewing the medical records and guaranteeing patient anonymity. Variables were of five types: 1—maternal characteristics: maternal age, number of gestations, parity, prepregnancy weight, maternal height, body mass index (BMI), smoking, gestational age (GA) at admission, and poor response to antihypertensive drugs; 2—maternal medical conditions: use of reproductive techniques, thrombophilia, diabetes; 3—maternal blood analysis: aspartate aminotransferase (AST), alanine transaminase (ALT), lactate dehydrogenase (LDH), platelets, creatinine, soluble fms-like tyrosine kinase-1 (S-Flt-1), placental growth factor (PlGF), S-Flt-1/PlGF, proteinuria, hemolysis, elevated liver enzymes, and low platelets (HELLP) syndrome; 4—maternal symptoms: headache, edema, photopsia, epigastric pain, dyspnea, dizziness; 5—fetal examination: estimated fetal weight (EFW) centile, umbilical artery pulsatility index (UA PI), middle cerebral artery pulsatility index (MCA PI), cerebroplacental ratio (CPR), multiples of the median (MoM), absent or reversed ductus venosus (DV), diagnosis of severe intrauterine growth restriction (IUGR), fetal sex, and abnormal cardiotocogram (CTG). Both the analysis and the ultrasound were performed at admission. On occasions, the ultrasound was delayed up to one day pending a higher definition ultrasound.

Finally, information about labor was also collected to determine the outcome. This included GA at labor, type of labor onset, mode of delivery, birth weight, Apgar score, arterial and venous pH, and neonate destiny (maternal ward, neonatal ward, intensive care unit (ICU), and morgue). We chose perinatal death as the dependent variable for the multivariable analysis.

To evaluate the relationship between the above-mentioned parameters and perinatal death, an univariable regression analysis was initially performed to select plausible determinants. Afterward, a multivariable regression analysis was carried out to create different models that were evaluated considering the area under the curve (AUC) and the Akaike Information Criteria (AIC). In these models, the AIC and the AUC with their 95% confidence interval (CI), *p*-value, and detection rates (DR) for a false positive rate (FPR) of 5% and 10% were calculated. Statistics and graphs were created using Graph Pad Prism 9^®^ and Stat Plus Pro 7^®^ for Apple Macintosh. Statistical significance was set at *p* < 0.05.

## 3. Results

### 3.1. Descriptive Analysis of the Population

One hundred and forty-eight patients were included in the study. Table 1 shows the descriptive analysis of the population in terms of mean, standard deviation (SD), median, and quartiles (Q) for the continuous parameters and in terms of numbers and percentages for the categorical ones. Two columns were constructed describing the perinatal death (N = 18) and the live fetuses (N = 130) population characteristics, as well as the comparison between them.

Most pregnant women were nulliparous (72.3%) and non-smokers (94.6%). The rate of pregnancies achieved with reproductive techniques was notorious (27%). Regarding clinical manifestations, the most frequent was headache (33.8%), followed by epigastric pain (24.3%) and photopsia (8.7%). HELLP syndrome occurred in only a small percentage (7.4%), and 60.8% of patients presented poor responses to antihypertensive drugs.

The mean maternal age was 35 years old and the mean prepregnancy weight and BMI were 73.9 kg and 27.9 kg/m^2^. Most fetuses were male (58.8%) and had low birth weight centiles (mean 9.9). In fact, 24.3% presented with severe intrauterine growth restriction (IUGR). All the deliveries were preterm (<37 weeks) (an inclusion criterion), and 70.3% were severe preterm (<34 weeks). Cesarean section (CS) was the main mode of delivery (91.9%), being elective in most cases (81.7%). No spontaneous onset of labor occurred. Low arterial pH was recorded in 14.9%, and a low Apgar score in 8.7% of the newborns. ICU was needed by 52.7% of them, while perinatal deaths (the dependent variable chosen for the outcome) occurred in 12.2% of the studied cases.

The comparison between the population with perinatal death (n = 18) and the live fetuses (n = 130) (Table 1) was significant in terms of all the parameters related to GA, Doppler study (both UA and ACM and, consequently, CPR), as well as birth weight and fetal sex. Regarding analytical parameters, only PlGF and s-Flt-1/PlGF ratio were significant. No clinical parameter showed differences between the two groups. The significance levels are expressed in the last column of Table 1.

### 3.2. Univariable Logistic Regression for the Prediction of Perinatal Death

Univariable logistic regression, as shown in Table 2, selected as significant the following: GA at admission, the mother’s poor response to antihypertensive drugs, PlGF, CPR MoM, UA PI, absent/reversed DV, and fetal sex [Table 2].

### 3.3. Multivariable Model

**Figure 1:**
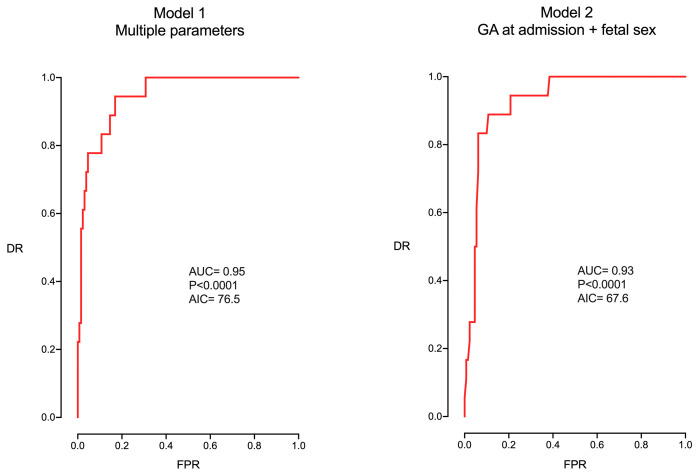
ROC curves of the multivariable models.

The multivariable model created with these parameters [Model 1 in Table 3 and Figure 1] presented high accuracy (AUC 0.95, AIC 76.5). However, the model that included only GA and fetal sex, the most important parameters in the previous model [Model 2 in Table 3 and Figure 1], presented a similar ability with a higher simplicity and reproducibility (lower AIC) (AUC 0.93, AIC 67.6).

**Figure 2:**
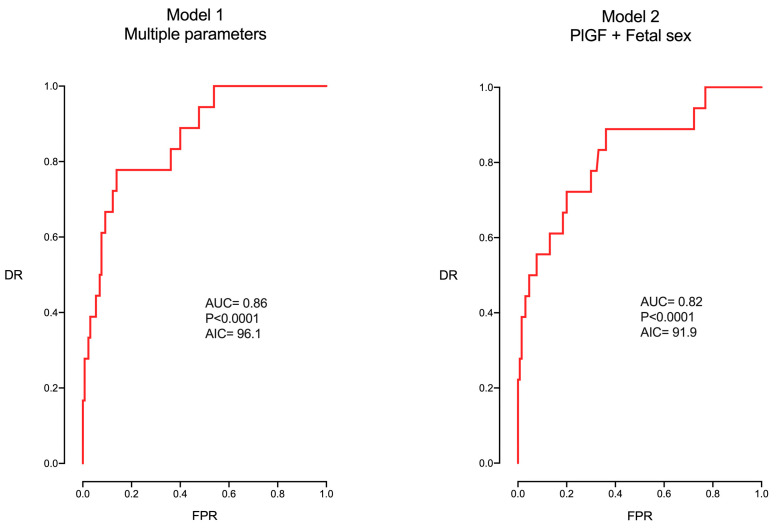
ROC curve of the multivariable model without including gestational age.

This procedure was repeated without including GA in the equation, mimicking a similar GA for all studied patients. In this case, the model [Model 1 in Table 4 and Figure 2] became less accurate (AUC 0.86, AIC 96.12), while fetal death became only dependent on PlGF and fetal sex. Again, the model that included only these two parameters presented a similar ability with a higher level of simplicity (AUC 0.82, AIC 91.9).

### 3.4. Effect Size of GA, PlGF, and Sex

**Figure 3:**
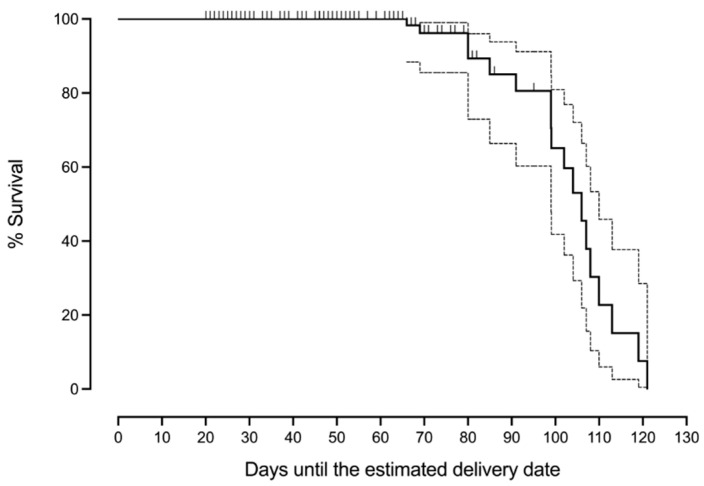
The Kaplan–Meier curve represents the percentage of fetal survival in the days until the estimated delivery date.

**Figure 4:**
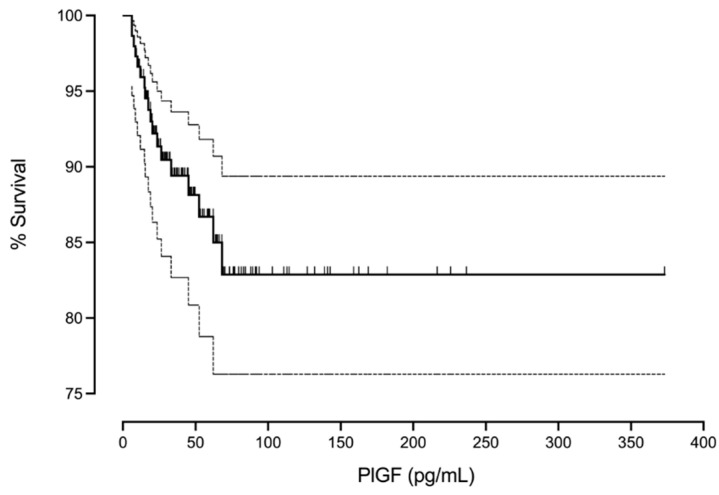
The Kaplan–Meier curve represents the percentage of fetal survival and the PlGF value in pg/mL.

**Figure 5:**
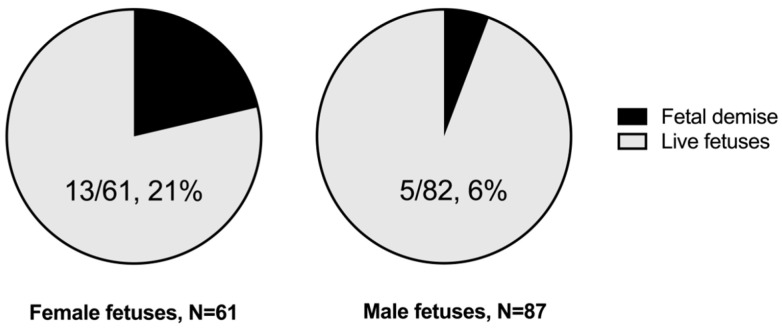
Pie chart representing the percentage of female and male fetuses among the live fetuses and the fetal demise.

To illustrate the effect size of GA, fetal sex, and PlGF, Figure 3, Figure 4 and Figure 5 were constructed.

Figure 3 represents the percentage of fetal survival according to the GA at birth (the X axis represents the days remaining until the estimated delivery date). It shows how survival decreases with low GA. A small drop in survival might be seen below 26 weeks (about 100 days before the estimated delivery date).

Figure 4 represents the percentage of fetal survival according to the values of PlGF. Low PlGF values are associated with less fetal survival. An increase in mortality might be seen with PlGF values below 75 pg/mL.

The percentage of each sex among the live and dead fetuses is represented in Figure 5. It shows that 21% of the female fetuses died, in contrast to only 6% of the male fetuses. In fact, despite the diagnosis of preterm PE being more frequent in male than female fetuses (our sample of preterm PE included 61 females and 82 males), female fetuses died between 3 and 4 times more than male fetuses.

## 4. Discussion

The appropriate time to terminate a pregnancy due to the development of PE remains a challenge in clinical practice. The maternal risk of continuing the pregnancy must be balanced against the newborn prognosis.

Multiple studies demonstrate the existence of a worse neonatal prognosis with low GA due to the extreme prematurity [21,22]. This study is consistent with this evidence and shows that GA at birth is the major determinant of neonatal death [23]. The decrease in survival is a continuum that slightly deepens below 26 weeks (Figure 3). A few combinations have been attempted to improve the prediction of GA for the risk of neonatal death, such as the conjunction of GA and the Apgar score [23], a postnatal parameter. However, to the best of our knowledge, up to now there is no accurate published prediction model for perinatal death in preterm PE.

Sex differences in cardiovascular risk throughout adult life are well known, as sex chromosomes and sex hormones influence blood pressure regulation, the distribution of cardiovascular risk factors, and other comorbidities [24,25,26]. Studies in pregnant rats have shown a different response to prenatal hypoxic stress according to sex via reducing the modulation of nitric oxide in females and increasing expression of thromboxane A2 in males, concluding that complicated pregnancies may lead to a sex difference in the programming of cardiovascular disease in adult offspring [27]. Furthermore, it is suggested there is sexual dimorphism in the feto-placental units, although its clinical relevance remains unknown [28]. A study shows higher levels of sFlt-1 and sFlt-1/PlGF ratio in pregnancies with female fetuses compared with male fetuses both in healthy pregnancies and in women who developed PE. However, a previous study had shown an increased level of sFlt-1 only in first-trimester normal gestations female fetuses, not in gestations that developed PE [29]. Although their results were not concordant, both suggest that fetal sex should be taken into account in the interpretation of angiogenic markers [28]. This study shows a clear and simple model for the prediction of perinatal death in preterm PE, including fetal sex and GA, with a very high prognostic power (AUC 0.93, AIC 67.6). Furthermore, there is a study that suggests a worse prognosis for male fetuses, possibly due to a higher rate of prematurity and IUGR compared with females, although with opposite results for gestations developing PE [30].

Of note, our results show the diagnosis of preterm PE was higher in male fetuses, but strikingly, females died 3.5 times more. To the best of our knowledge, this is a novel finding that launches a hypothesis to be corroborated by larger sample studies.

Regarding the PlGF values, our results show a lower survival with lower values of PlGF, especially below 75 pg/mL (Figure 4). It is known the PlGF values vary with GA [31], and accordingly, it could be argued that in our study the reason for the low PlGF was the low GA, making the use of PlGF MoM mandatory. However, the PlGF normality curve remains very stable around the weeks corresponding to our study period [30]. In addition, in the multivariable analysis we adjusted PlGF for GA, and both GA and PlGF were selected as significant parameters, discarding any type of interaction between both variables, which seem to be independent (Table 3).

Furthermore, for the same GA, the best parameters were PIGF and fetal sex, predicting fetal demise with an AUC of 0.82 and an AIC of 91.90.

These results show a different prognosis in neonates of gestations with PE according to sex, with a higher rate of PE diagnoses in male fetuses but a higher rate of fetal demise in female fetuses. Moreover, at the same GA, fetal death also becomes dependent on PlGF, an influence that seems independent of GA. Although further studies are needed, this finding might be the seed for a new approach to PE management.

The authors acknowledge some limitations of this study, such as the small sample size for the outcome variable (perinatal death, n = 18) and the lack of information in collecting clinical history data due to the study’s retrospective design. Although the study was developed in a center with homogeneous management of clinical cases, more studies with prospective designs are necessary for internal and external validation of the results.

## 5. Conclusions

Fetal sex and PlGF are notorious predictors of perinatal death in preterm preeclampsia, only surpassed by GA at birth.

## Figures and Tables

**Table 1:** Description of the study population (N = 148).

	1-All Cases (N = 148)		2-Perinatal Death (n = 18)		3-Live Fetuses (n = 130)		(2 versus 3)
Continuous Parameters	Mean (SD)	Median (1st, 3rd Q)	Mean (SD)	Median (1st, 3rd Q)	Mean (SD)	Median (1st, 3rd Q)	*p*-Value
Maternal age	34.9 (6.1)	35 (31, 39)	34 (6.9)	35 (30.5, 39)	35 (6)	35 (31, 39)	0.7585
Number of gestations	2.0 (1.3)	1.5 (1, 3)	1.4 (0.7)	1 (1, 2)	2.1 (1.4)	2 (1, 3)	0.0704
Parity	0.5 (0.9)	0 (0, 1)	0.1 (0.3)	0 (0, 0)	0.51 (0.94)	0 (0, 1)	0.1955
Prepregnancy weight (kg)	73.9 (17.1)	71.7 (61.4, 84.9)	79.6 (17.7)	76 (63.5, 90)	73.1 (17)	70.9 (60, 83.6)	0.1196
Height (cm)	162.0 (6.9)	158 (162, 165)	163.7 (6.5)	163 (159, 170)	161.8 (6.9)	162 (158, 165)	0.2655
BMI (kg/m^2^)	27.9 (6.1)	27.1 (23.1, 31.1)	29.7 (6.3)	27.2 (24.75, 33.3)	27.7 (6.1)	27.1 (23, 31)	0.2351
GA at admission	30.9 (3.8)	31.3 (28.3, 34.0)	25.6 (2.3)	25.1 (23.9, 27)	31.6 (3.3)	32.1 (29.1, 34.4)	<0.0001
GA at examination (weeks)	31.25 (3.6)	31.9 (28.8, 34)	25.8 (2.4)	25.5 (23.8, 27.4)	32 (3)	32.6 (30, 34.6)	<0.0001
GA at labor	31.7 (3.5)	32.4 (29.5, 34.6)	26 (2.3)	25.6 (24.5, 28)	32.5 (2.8)	33 (30.3, 35)	<0.0001
EFW in grams	1432 (639.5)	1373 (945, 1909)	653 (333)	579 (438, 750)	1539 (596)	1464 (1068, 1956)	<0.0001
EFW centile	12.6 (24.4)	1 (0, 10.75)	5.4 (20.9)	1 (0, 1)	13.6 (24.6)	2 (0, 13)	0.0031
UA PI	1.5 (0.61)	1.3 (1.07, 1.99)	2.03 (0.5)	2.3 (1.4, 2.4)	1.44 (0.58)	1.23 (1.04, 1.68)	<0.0001
MCA PI	1.50 (0.41)	1.44 (1.16, 1.75)	1.34 (0.46)	1.21 (1.04, 1.68)	1.52 (0.4)	1.46 (1.20, 1.80)	0.0348
CPR	1.17 (0.57)	1.18 (0.64, 1.60)	0.74 (0.43)	0.55 (0.47, 0.79)	1.23 (0.56)	1.27 (0.73, 1.64)	0.0002
UA PI MoM	1.5 (0.6)	1.31 (1.07, 1.99)	1.89 (0.45)	2.05 (1.43, 2.13)	1.58 (0.60)	1.37 (1.15, 1.95)	0.0060
MCA PI MoM	1.50 (0.41)	1.44 (1.16, 1.75)	0.67 (0.24)	0.61 (0.50, 0.83)	0.77 (0.20)	0.75 (0.63, 0.88)	0.0361
CPR MoM	1.17 (0.57)	1.18 (0.64, 1.60)	0.41 (0.25)	0.33 (0.27, 0.44)	0.62 (0.28)	0.63 (0.37, 0.81)	0.0019
Interval admission-delivery (days)	5.5 (9.7)	3 (1, 6)	2.6 (3.5)	1.5 (0, 5.25)	5.89 (10.2)	3 (1, 6.2)	0.0496
AST (GOT)	56.1 (96.9)	24.5 (18, 43)	61.7 (93.6)	25 (17.5, 71.5)	55.3 (97.7)	24 (17.75, 43)	0.7301
ALT (GPT)	57.8 (106.6)	20 (13.2, 50.2)	64.7 (99.7)	24.5 (17.5, 86.7)	56.9 (107.8)	19 (13, 48.7)	0.2954
LDH	277.8 (135.5)	250 (214.3, 295)	301 (95.7)	278 (226, 364)	274.6 (140.1)	244 (211.8, 292.3)	0.0636
Platelets × 1000	196.16 (64.22)	192.5 (157.5, 233)	201.4 (96)	202 (138, 234)	195.4 (58.9)	192 (158.5, 233.2)	0.8580
Creatinine	0.71 (0.24)	0.58 (0.66, 0.79)	0.76 (0.51)	0.66 (0.56, 0.73)	0.71 (0.18)	0.66 (0.59, 0.79)	0.3954
s-Flt-1	15,203 (8353)	13,025 (9888, 17,961)	15,219 (5581)	13,343 (11,037, 17,656)	15,201 (8683)	13,025 (9563, 18,088)	0.5313
PlGF	55.89 (51.1)	41.9 (22.5, 69)	24.95 (19.5)	18.3 (9.6, 36.2)	60.2 (52.6)	45.8 (25.5, 74.1)	<0.0001
s-Flt-1/PlGF	953.9 (4408)	347.5 (159, 737)	1001 (654)	1025 (434, 1606)	947.3 (4700)	325 (150, 712)	0.0003
Proteinuria	201.9 (295.1)	100 (0, 300)	212 (315)	100 (22.5, 300)	200.6 (293.5)	100 (0, 300)	0.7378
Birth weight in grams	1432 (671)	1318 (992, 1798)	631 (325)	565 (404, 800)	1543 (630)	1415 (1158, 1863)	<0.0001
Birth weight centile	9.9 (24)	0 (0, 5)	3.9 (15)	0 (0, 0)	10.7 (24.8)	1 (0, 5)	0.0088
Categorical parameters	N (%)		N (%)		N (%)		*p*-value
Perinatal deaths	18 (12.2)		18 (100)		0 (0)		<0.0001
Fetal sex (male)	87 (58.8)		5 (27.8)		82 (63.1)		0.0089
Smoking	8 (5.4)		0 (0)		0 (0)		1
Headache	50 (33.8)		5 (27.8)		45 (34.6)		0.7909
Edema	27 (18.2)		2 (11.1)		25 (19.2)		0.5290
Photopsia	13 (8.7)		2 (11.1)		11 (8.5)		0.6600
Epigastric pain	36 (24.3)		1 (5.5)		35 (26.9)		0.0744
Dyspnea	2 (1.3)		0 (0)		2 (1.5)		1
Dizziness	6 (4)		0 (0)		6 (4.6)		1
Diagnosis of HELLP syndrome	11 (7.4)		3 (16.7)		8 (6.1)		0.1335
Absent or reversed UA	34 (23)		6 (33.3)		28 (21.5)		0.3807
Absent or reversed Ductus Venosus	4 (2.7)		2 (11.1)		2 (1.5)		0.0391
Diagnosis of severe IUGR	30 (20.3)		4 (22.2)		26 (20)		0.7621
Abnormal CTG	36 (24.3)		5 (27.8)		31 (23.8)		0.7708
Poor response to antihypertensive drugs	90 (60.8)		7 (38.9)		83 (63.8)		0.0688
Reproductive techniques (any)	40 (27)		4 (22.2)		36 (27.7)		0.7805
IVF Ovodon	27 (18.2)		2 (11.1)		25 (19.2)		0.5290
Thrombophilia	3 (2)		0 (0)		3 (2.3)		1
Diabetes	24 (16.2)		0 (0)		24 (18.5)		0.0450
Nulliparity	107 (72.3)		15 (83.3)		92 (70.8)		0.4001
Severe preterm labor incidence (<34)	104 (70.3)		18 (100)		86 (66.1)		0.0017
Preterm labor incidence (<37 weeks)	148 (100)		18 (100)		128 (98.4)		0.0150
Type of labor onset							
Cesarean section (elective)	121 (81.7)		12 (66.7)		109 (83.8)		0.1007
Induction of labor	3 (2)		3 (16.7)		21 (16.1)		1
Spontaneous onset of labor	24 (16.2)		3 (16.7)		0 (0)		0.0015
Apgar < 7 at 5 min	13 (8.7)		10 (55.5)		3 (2.3)		<0.0001
Arterial pH < 7.10	22 (14.9)		6 (33.3)		4 (3.1)		0.0002
Via of delivery							
CS (elective)	121 (81.7)		12 (66.7)		109 (83.8)		0.1007
CS (failure to progress)	3 (2)		0 (0)		3 (2.3)		1
CS (abnormal CTG)	12 (8.1)		2 (11.1)		10 (7.7)		0.6413
Assisted vaginal delivery	1 (0.7)		0 (0)		1 (0.8)		1
Spontaneous vaginal delivery	11 (7.4)		4 (22.2)		7 (5.4)		0.0278
Neonate destiny							
Maternal ward	12 (8.1)		0 (0)		12 (9.2)		0.3619
Neonates ward	51 (34.4)		0 (0)		51 (39.2)		0.0004
Pediatric Intensive care unit	78 (52.7)		11 (61.1)		67 (51.5)		0.6155
Morgue	7 (4.7)		7 (38.9)		0 (0)		<0.0001

**Notes**: SD: standard deviation, Q: quartiles, CS: cesarean section, BMI: body mass index, GA: gestational age, EFW: estimated fetal weight, BW: birth weight, UA: umbilical artery, MCA: middle cerebral artery, PI: pulsatility index, CPR: cerebroplacental ratio, MoM: multiples of median, s-Flt-1: soluble fms-like tyrosine kinase 1, PLGF: placental growth factor, AST: aspartate aminotransferase, ALT: alanine transaminase, LDH: lactate dehydrogenase, HELLP: hemolysis, elevated liver enzymes, and low platelets, IVF: in vitro fertilization, CTG: cardiotocogram, IUGR: intrauterine growth restriction.

**Table 2:** Univariable logistic regression for the prediction of perinatal death.

Parameter	Estimate	SE	OR (95% Confidence Interval)	*p*-Value
Maternal characteristics				
Maternal age	−0.026	0.040	0.974 (0.900, 1.055)	0.524
Gestations	−0.555	0.302	0.574 (0.317, 1.039)	0.067
Parity	−0.717	0.502	0.488 (0.182, 1.305)	0.153
Prepregnancy weight (kg)	0.020	0.014	1.020 (0.994, 1.048)	0.133
Height	0.042	0.037	1.043 (0.970, 1.121)	0.258
BMI	0.050	0.038	1.051 (0.975, 1.133)	0.194
Smoking	−14.279	660.925	0.000 (0.000, N/A)	0.983
Gestational age at admission	−0.570	0.120	0.566 (0.447, 0.716)	<0.0001
Poor response to Anti-HT	−1.021	0.516	0.360 (0.130, 0.992)	0.048
Maternal medical conditions				
Reproductive techniques	−0.293	0.600	0.746 (0.230, 2.417)	0.625
Thrombophilia	−13.230	694.329	0 (0, N/A)	0.985
Diabetes	−15.411	667.290	0 (0, N/A)	0.981
Maternal analysis				
AST	0.002	0.002	1.000 (0.995, 1.005)	0.794
ALT	0.000	0.002	1.000 (0.996, 1.004)	0.769
LDH	1.001	0.001	1.001 (0.998, 1.004)	0.450
Platelets	0.000	0.000	1.000 (1.000, 1.000)	0.712
Creatinine	1.975	0.809	1.975 (0.405, 9.641)	0.400
s-Flt-1	0.000	0.000	1.000 (1.000, 1.000)	0.993
PlGF	−0.049	0.016	0.952 (0.922, 0.983)	0.003
Ratio s-Flt-1/PlGF	0.000	0.000	1.000 (1.000, 1.000)	0.961
Proteinuria	0.000	0.000	1.000 (0.998, 1.001)	0.881
Diagnosis of HELLP syndrome	1.1115	0.730	3.050 (0.729, 12.760)	0.126
Maternal symptoms				
Headache	−0.319	0.557	0.726 (0.243, 2.167)	0.566
Edema	−0.644	0.782	0.525 (0.113, 2.433)	0.410
Photopsia	0.302	0.813	1.352 (0.274, 6.661)	0.711
Epigastric pain	−1.835	1.048	0.160 (0.020, 1.244)	0.080
Dyspnea	−13.223	850.386	0.000 (0.000, N/A)	0.987
Dizziness	−14.254	809.464	0.000 (0.000, N/A)	0.986
Fetal examination				
EFW centile	−0.020	0.020	0.980 (0.942, 1.019)	0.310
UA PI MoM	0.745	0.379	2.107 (1.003, 4.425)	0.049
MCA PI MoM	−2.458	1.430	0.086 (0.005, 1.440)	0.088
CPR MoM	−3.232	1.174	0.039 (0.004, 0.394)	0.006
Absent or reversed UA flow	0.600	0.543	1.820 (0.627, 5.2869)	0.270
Absent/Reversed DV	2.079	1.034	8.000 (1.053, 60.775)	0.044
Diagnosis of severe IUGR	0.133	0.607	1.143 (0.347, 3.762)	0.826
Fetal sex	−1.491	0.557	0.225 (0.076, 0.670)	0.007
Abnormal CTG	0.205	0.565	1.228 (0.406, 3.718)	0.716

**Notes**: BMI: body mass index, SE: standard error, OR: Odds ratio, EFW: estimated fetal weight, UA PI: umbilical artery pulsatility index, CPR: cerebroplacental ratio, MoM: multiples of median, DV: ductus venosus, s-Flt-1: soluble fms-like tyrosine kinase 1, PLGF: placental growth factor, AST: aspartate aminotransferase, ALT: alanine transaminase, LDH: lactate dehydrogenase, HELLP: hemolysis, elevated liver enzymes, and low platelets, IUGR: intrauterine growth restriction, CTG: cardiotocogram.

**Table 3:** Multivariable logistic models for the prediction of perinatal death.

	Estimate	SE	OR (95% CI)	*p*-Value
**Model 1** (Parameters with significance or borderline significance in the univariable analysis.)				
Gestations	−0.60941	0.40826	0.54367 (0.24424, 1.21021)	0.13552
GA at admission	−0.54820	0.14787	0.57799 (0.43256, 0.77231)	0.00021
Poor response to anti-HTA drugs	0.37492	0.93699	1.45488 (0.23186, 9.12872)	0.68906
PlGF	−0.00944	0.01849	0.99060 (0.95535, 1.02716)	0.60961
Epigastric pain	−2.02249	1.29514	0.13233 (0.01045, 1.67529)	0.11838
UA PI MoM	−0.22253	0.75392	0.80049 (0.18265, 3.50836)	0.76787
MCA PI MoM	−0.09211	2.08763	0.91201 (0.01524, 54.57851)	0.96481
Absent or rev DV	0.89366	1.72898	2.44405 (0.08248, 72.41841)	0.60525
Fetal sex	−1.42443	0.75276	0.24064 (0.05503, 1.05230)	0.05845
Intercept	16.01822			
AIC: 76.5, AUC: 0.95, 95% CI (0.90–0.99), *p* < 0.0001, DR 78% for a FPR of 5%, DR 78% for a FPR of 10%.				
**Model 2** (Parameters with significance or borderline significance in the multivariable analysis.)				
GA at admission	−0.55584	0.12091	0.5735 (0.4525, 0.7269)	*p* < 0.0001
Fetal sex	−1.37249	0.68601	0.2534 (0.0660, 0.9724)	0.04543
Intercept	14.19035			
AIC: 67.6, AUC: 0.93, 95% CI (0.88–0.98), *p* < 0.0001, DR 50% for a FPR of 5%, DR 83% for a FPR of 10%.				

**Notes**: GA: gestational age, OR: odds ratio, MoM: multiples of the median, UA PI: umbilical artery pulsatility index, CPR: cerebroplacental ratio, MoM: multiples of median, DV: ductus venosus, CI: confidence interval, AIC: Akaike Information Criterion, AUC: area under the curve, DR: discrimination rate, FPR: false positive rate, SE: standard error, CI: confidence interval.

**Table 4:** Multivariable logistic models for the prediction of perinatal death in fetuses with the same GA (In this case, GA was excluded).

	Estimate	SE	OR (95% CI)	*p*-Value
Model 1 (Parameters with significance or borderline significance in the univariable analysis, excluding GA at admission.)				
Gestations	−0.48439	0.32905	0.61607 (0.32325, 1.17416)	0.14100
Poor response to anti-HTA drugs	−0.67533	0.73514	0.50899 (0.12049, 2.15017)	0.35828
PlGF	−0.04605	0.01743	0.95499 (0.92291, 0.98818)	0.00824
Epigastric pain	−1.60225	1.17818	0.20144 (0.02001, 2.02788)	0.17385
UA PI MoM	−0.03551	0.56005	0.96512 (0.32200, 2.89272)	0.94945
MCA PI MoM	0.15408	1.65317	1.16658 (0.04568, 29.79332)	0.92574
Absent or reverse DV	1.00759	1.43756	2.73899 (0.16365, 45.84237)	0.48336
Fetal sex	−1.41739	0.62826	0.24235 (0.07074, 0.83027)	0.02407
Intercept	1.62196			
AIC: 96.12. AUC: 0.86. 95% CI (0.78–0.95). *p* < 0.0001. DR 38% for a FPR of 5%. DR 67% for a FPR of 10%.				
Model 2 (parameters with significance or borderline significance in the multivariable analysis, excluding GA at admission)				
PlGF	−0.04713	0.01618	0.95397 (0.92419, 0.98470)	0.00358
Fetal sex	−1.46315	0.58312	0.23151 (0.07382, 0.72597)	0.01210
Intercept	0.39035			
AIC: 91.90, AUC: 0.82, 95% CI (0.71–0.93), *p* < 0.0001, DR 50% for a FPR of 5%, DR 55% for a FPR of 10%.				

**Notes**: GA: gestational age, OR: odds ratio, MoM: multiples of the median, UA PI: umbilical artery pulsatility index, CPR: cerebroplacental ratio, MoM: multiples of median, DV: ductus venosus, CI: confidence interval, AIC: Akaike Information Criterion, AUC: area under the curve, DR: discrimination rate, FPR: false positive rate, SE: standard error, CI: confidence interval.

## Data Availability

Data available on request from the authors.

## Abbreviations

PE	Preeclampsia
SD	Standard deviation
Q	Quartiles
BMI	Body Mass Index
GA	Gestational age
EFW	Estimated fetal weight
BW	Birth weight
AST	Aspartate aminotransferase
ALT	Alanine transaminase
LDH	Lactate dehydrogenase
Hemolysis, elevated liver enzymes, and low platelets HELLP syndrome	
S-Flt-1	Soluble fms-like tyrosine kinase-1
PlGF	Placental growth factor
UA	Umbilical artery
MCA	Middle cerebral artery
PI	Pulsatility index
CPR	Cerebroplacental ratio
MoM	Multiples of the median
IVF	In vitro fertilization
CTG	Cardiotocogram
IUGR	Intrauterine growth restriction
ICU	Intensive care unit
CS	Cesarean section
